# A critical appraisal of ferroptosis in Alzheimer’s and Parkinson’s disease: new insights into emerging mechanisms and therapeutic targets

**DOI:** 10.3389/fphar.2024.1390798

**Published:** 2024-07-08

**Authors:** Priyanka Soni, Navneet Ammal Kaidery, Sudarshana M. Sharma, Irina Gazaryan, Sergey V. Nikulin, Dmitry M. Hushpulian, Bobby Thomas

**Affiliations:** ^1^ Darby Children’s Research Institute, Medical University of South Carolina, Charleston, SC, United States; ^2^ Department of Pediatrics, Medical University of South Carolina, Charleston, SC, United States; ^3^ Department of Biochemistry and Molecular Biology and Hollings Cancer Center, Medical University of South Carolina, Charleston, SC, United States; ^4^ Department of Chemical Enzymology, School of Chemistry, M.V. Lomonosov Moscow State University, Moscow, Russia; ^5^ Department of Chemistry and Physical Sciences, Dyson College of Arts and Sciences, Pace University, Pleasantville, NY, United States; ^6^ Faculty of Biology and Biotechnologies, Higher School of Economics, Moscow, Russia; ^7^ A.N.Bach Institute of Biochemistry, Federal Research Center “Fundamentals of Biotechnology” of the Russian Academy of Sciences, Moscow, Russia; ^8^ Department of Neuroscience, Medical University of South Carolina, Charleston, SC, United States; ^9^ Department of Drug Discovery, Medical University of South Carolina, Charleston, SC, United States

**Keywords:** ferroptosis, iron homeostasis, lipid peroxidation, neurodegenerative diseases, Nrf2

## Abstract

Neurodegenerative diseases represent a pressing global health challenge, and the identification of novel mechanisms underlying their pathogenesis is of utmost importance. Ferroptosis, a non-apoptotic form of regulated cell death characterized by iron-dependent lipid peroxidation, has emerged as a pivotal player in the pathogenesis of neurodegenerative diseases. This review delves into the discovery of ferroptosis, the critical players involved, and their intricate role in the underlying mechanisms of neurodegeneration, with an emphasis on Alzheimer’s and Parkinson’s diseases. We critically appraise unsolved mechanistic links involved in the initiation and propagation of ferroptosis, such as a signaling cascade resulting in the de-repression of lipoxygenase translation and the role played by mitochondrial voltage-dependent anionic channels in iron homeostasis. Particular attention is given to the dual role of heme oxygenase in ferroptosis, which may be linked to the non-specific activity of P450 reductase in the endoplasmic reticulum. Despite the limited knowledge of ferroptosis initiation and progression in neurodegeneration, Nrf2/Bach1 target genes have emerged as crucial defenders in anti-ferroptotic pathways. The activation of Nrf2 and the inhibition of Bach1 can counteract ferroptosis and present a promising avenue for future therapeutic interventions targeting ferroptosis in neurodegenerative diseases.

## 1 Introduction

Neurodegenerative diseases stand as enigmatic challenges within the scientific community, quietly disrupting the delicate orchestra of the human brain and causing gradual neuronal loss, impacting movement, cognition, and overall functionality. While the precise etiology of these diseases, encompassing a spectrum from Alzheimer’s to Parkinson’s and beyond, remains elusive, oxidative stress and mitochondrial dysfunction have long been implicated (Ji et al., 2022). In recent years, ferroptosis, a non-apoptotic cell death driven by lipid peroxidation, has emerged as a novel player in neurodegeneration. Ferroptosis derives its name from its iron dependency and the Greek origin “ptosis,” meaning “falling” (Lin et al., 2022). This form of regulated cell death is characterized by the production of toxic reactive oxygen species (ROS) and lipid peroxides in cell membranes, leading to alteration in the cell morphology, destabilization of the plasma membrane, cytoskeletal rearrangements, mitochondrial atrophy or fragmentation (condensed mitochondrial membrane density and decreased mitochondrial cristae), with no chromatin condensation and nuclear reduction, disruption of proteostasis, glutathione (GSH) depletion, and glutathione peroxidase 4 (GPX4) inactivation (Cao and Dixon, 2016; Dodson et al., 2019; Hirschhorn and Stockwell, 2019). It is distinct from apoptosis, where cells shrink and fragment, or necrosis, characterized by swelling and bursting. In 2018, the Nomenclature Committee on Cell Death defined ferroptosis as a Regulated Cell Death (RCD) caused by oxidative distress in the intracellular microenvironment, which can be inhibited by lipophilic antioxidants and iron chelators (Sun et al., 2022).

Ferroptosis, initially observed and described as a novel mechanism by Stockwell and colleagues in 2012 (Dixon et al., 2012) for erastin-induced death, is of great interest for the potential treatment of cancer. The literature search in PubMed shows that the number of publications on ferroptosis in cancer represents more than half of all publications on ferroptosis (>10,000 since 2012). In contrast, the number of publications linking ferroptosis and neurodegenerative diseases searched as combination of words including ferroptosis, and the name of various neurodegenerative disease is less than 10% of the total number published (∼800 papers only). Quite a few studies have shown the involvement of ferroptosis in neurodegenerative diseases, and the core mechanisms revolve around three key components: iron, lipid peroxidation, and the antioxidant system (Latunde-Dada, 2017; Viktorinova and Durfinova, 2021). In this review, we intend to pinpoint the gaps in our knowledge of the detailed mechanism of ferroptosis initiation and propagation and discuss the potential benefits of activating Nrf2 and inhibiting the Bach1 signaling pathway for counteracting ferroptosis in neurodegenerative diseases.

## 2 Mechanisms of ferroptosis initiation and propagation

**FIGURE 1:**
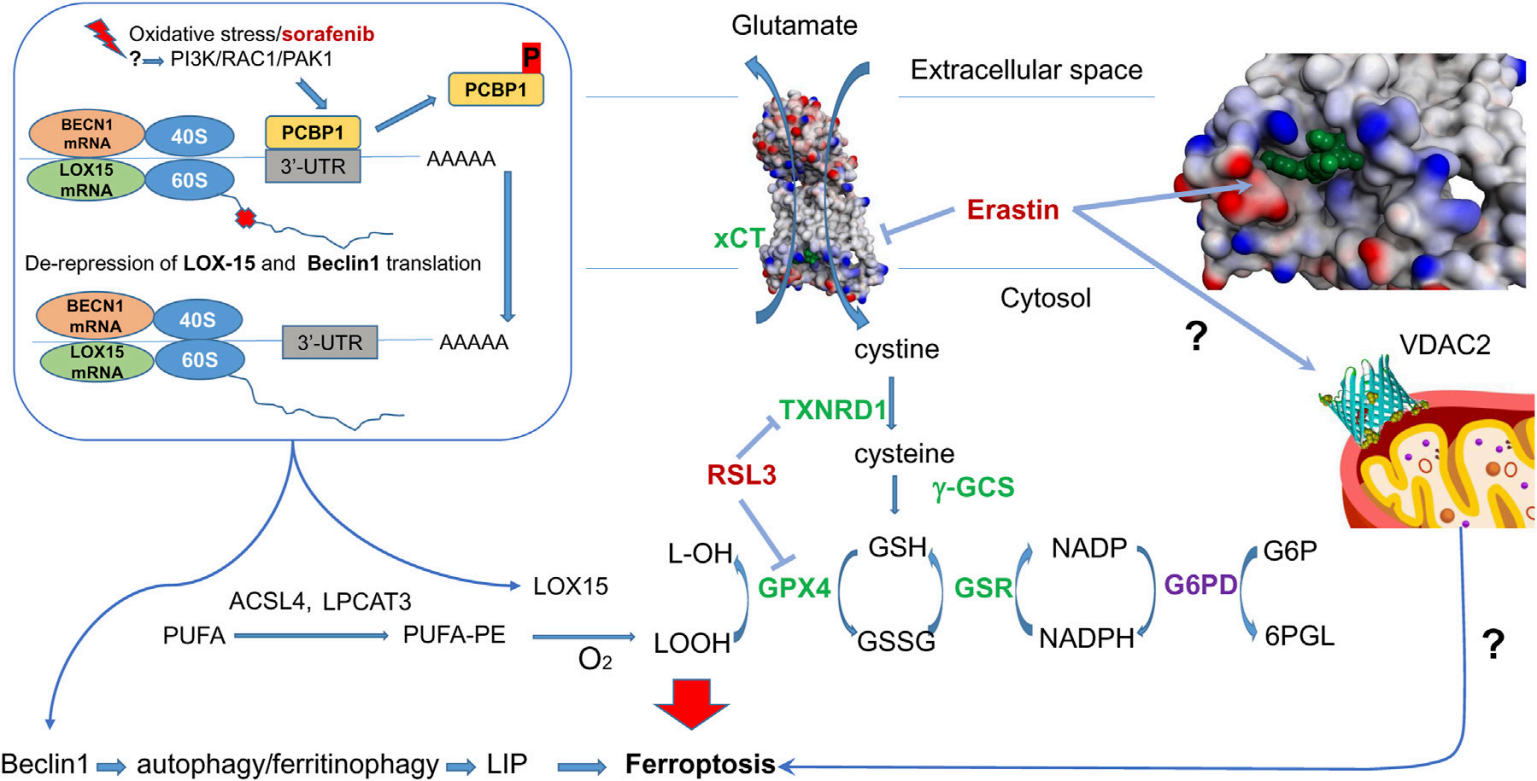
**Erastin and RSL3 targets in Ferroptosis**. Erastin inhibits cysteine-glutamate antiporter - erastin bound in xCT-4F2hc complex (7EPZ.pdb), shown in green–and targets mitochondrial VDAC2. In the absence of ferroptosis inducers, cystine entering the cell is converted into cysteine, then is used for GSH synthesis with gamma-glutamyl-cysteine synthetase (gGCS). GPX4 reduces lipid peroxides formed from polyunsaturated fatty acids (PUFA) with reduced glutathione (GSH), regenerated with the help of glutathione reductase (GSR) and NADPH. The latter is produced by glucose-6-phosphate dehydrogenase (G6PD), a HIF target). RSL3 inhibits selenoenzymes–glutathione peroxidase 4 (GPX4) and thioredoxin reductase 1 (TXNRD1). In the presence of ferroptosis inducers, lipoxygenase (LOX15) is activated (see text for details). A mechanism of lipoxygenase activation proceeds via de-repression of LOX15 translation upon phosphorylation of PCBP1, which happens once the PI3K/RAC1/PAK1 kinase pathway is activated in response to ferroptosis inducers. In addition to lipoxygenase, de-repression is observed for Beclin 1, which is directly implicated in autophagy and ferritinophagy. Nrf2 target genes are shown in green.

Ferroptosis, initially discovered during the screening of small molecule pharmacophores for cancer treatment, has seen significant advances in mechanistic understanding in recent years. Among these compounds, erastin is a classical inductor of ferroptosis in various cell lines (Xie et al., 2016). Investigating the targets of erastin has revealed a crucial link between cysteine metabolism and the initiation of ferroptosis (Xu et al., 2019). The primary mechanism through which erastin induces ferroptosis involves its interaction with the cystine-glutamate antiporter system Xc–, explicitly targeting the subunit xCT among the two subunits (light chain xCT and the heavy chain 4F2hc encoded by the SLC7A11 and SLC3A2 genes, respectively). Erastin thus restricts the access of cystine into the cytosol for its subsequent reduction into cysteine, followed by the synthesis of glutathione (Figure 1). The elucidation of the actual structure of the complex with erastin (Yan et al., 2022) opens the prospects for the design of drugs explicitly targeting this site.

Moreover, erastin also acts on voltage-dependent anion channel 2 (VDAC2) in the outer mitochondrial membrane (OMM). However, the precise mechanism by which erastin affects VDAC2 and its contribution to ferroptosis induction still needs to be fully understood. VDAC2 is known to regulate the flux of metabolites, ions, and small molecules between the cytosol and the mitochondria, suggesting its involvement in cellular metabolism and homeostasis. The interplay between these two mechanisms—erastin’s inhibition of the cystine-glutamate antiporter and its interaction with VDAC2—likely contributes to the subsequent activation of lipid peroxidation and iron imbalance, critical hallmarks of ferroptosis. However, the exact stepwise mechanism linking these events and their precise interconnections is still the subject of intense research. Mechanistically, erastin-induced cell death models two general features of neurodegeneration–mitochondrial damage and progressive oxidation due to glutathione depletion. Therefore, we mainly focus in this review on consideration of fine mechanistic details of erastin-induced cell death pathways and the potential players involved.

### 2.1 The build-up of lipoxygenase protein upon induction of ferroptosis

Glutathione depletion induced by erastin leads to a decreased activity of a selenium enzyme, GPX4, which neutralizes lipid peroxides. GPX4 catalyzes the reduction of lipid hydroperoxides (LOOH) to the corresponding alcohols (LOH) with reduced glutathione (GSH), whose regeneration from the oxidized glutathione is provided by NADPH-dependent glutathione reductase. The inhibition or depletion of GPX4 is central to the initiation of ferroptosis as its function relies on the oxidation of the vital cellular antioxidant, GSH. Direct inhibition of GPX4 by RAS-selective lethal (RSL3) exerts a more potent effect than erastin. Within 8 and 4 h of the treatment by 10 μM erastin and 1 μM RSL3, respectively, an increase in lipoxygenase enzymes (specifically LOX-12 and LOX-15) is seen by immunoblotting (Shintoku et al., 2017). This suggests that both treatments push the cells to make more of these enzymes by activating their expression or enhancing their translation. Of note, a similar phenomenon was observed long before the term “ferroptosis” was coined in a glutathione depletion model induced by glutamate in immature neurons, resulting in a 3-fold increase in LOX-12 protein detected at 8 h of incubation (see Figure 3C in (Li et al., 1997)). Thus, it is not surprising, given the substantial similarity in the hallmarks of ferroptosis observed both in cancer cell lines and immature neurons, as reported recently (Zille et al., 2019).

The exact mechanism leading to this phenomenon is yet to be determined. Still, we can speculate that this effect is related to the change in the functioning of an iron chaperone Poly (rC)-binding protein (PCBP1). It binds iron from the chemically reactive, labile iron pool (LIP) in the cytosol and delivers it to ferritin and non-heme iron enzymes via direct, metal-mediated protein-protein interactions. PCBP1, independent of its iron-binding activity (Patel et al., 2021), represses the translation of 15-lipoxygenase (Lee et al., 2022) (Figure 1). The transcriptional and translational repressing activity of PCBP1 is controlled by phosphorylation. When another ferroptosis inducer, sorafenib (an FDA-approved multi-kinase inhibitor), is added, the phosphorylation of PCBP1 changes within 60 min (Werth et al., 2020). Hence, we may speculate that at the ferroptosis initiation step, there has to be a change in PCBP1 phosphorylation that results in the de-repression of LOX-15 translation on the ribosome and the production of more LOX protein. Of note, PCBP1 not only regulates LOX-15 but also inhibits autophagy by binding to the CU-rich elements on the 3′-untranslated region (3′-UTR) of Beclin 1 (BECN1) mRNA (Lee et al., 2022). Hence, de-repression of BECN1 will lead to iron-mediated ferritinophagy. In addition, AMPK-mediated BECN1 phosphorylation results in blocking system Xc- (Song et al., 2018). Although the de-repression of LOX-15 and BECN1 is likely to happen simultaneously, the activation of the LOX-15-catalyzed reaction will trigger ferroptosis initiation, whereas ferritinophagy will represent the later propagation step. The translational repression of LOX-15 and BECN1 makes PCBP1 a seminal ferroptosis regulator by inhibiting iron-mediated ferritinophagy and lipid peroxidation (Figure 1).

A known kinase cascade involving PI3K-RAC1-PAK1 leads to the phosphorylation of PCBP1, eventually causing its degradation (Meng et al., 2007). However, the detailed pathway resulting in PCBP1 phosphorylation/dephosphorylation in ferroptosis initiation by various inducers requires further investigation. Another path to catalytic activation of LOX is well-documented for the LOX-12 enzyme, which is known to be activated by glutathionylation (Park et al., 2009), the process triggered by glutathione depletion, and by decreasing the ratio of reduced to oxidized glutathione.

### 2.2 Catalytic *versus* non-catalytic lipid peroxidation in ferroptosis

Lipid peroxidation stands as the significant characteristic and driving force of ferroptosis (Stockwell et al., 2020). This oxidative process can occur either enzymatically, driven by non-heme iron dioxygenases - lipoxygenases, as discussed above, or non-enzymatically (Ayala et al., 2014) through a radical chain reaction with likely participation of the ferrous-ion catalyzed reaction with hydrogen peroxide to generate hydroxyl-radical, the so-called Fenton reaction (Stockwell et al., 2020; Dang et al., 2022). The role of the Fenton reaction in lipid auto-oxidation is still debated. Polyunsaturated fatty acids (PUFAs) are especially prone to autoxidation because the C–H bonds of the methylene groups flanked by C-C double bonds are among the weakest C–H bonds known (ca. 76 kcal/mol). Increasing the number of double bonds in the lipid leads to a corresponding increase in the rate of its autoxidation (Conrad and Pratt, 2019).

Lipid hydroperoxides, specifically those of PUFAs, like arachidonoyl (AA) and adrenoyl (AdA) acids, induce severe damage to the lipids in the plasma membrane, leading to ferroptosis (Dang et al., 2022). The secondary oxidation products are aldehydes such as malondialdehyde (MDA) and 4-hydroxynonenal (4-HNE), which have even more damaging chemistry. AA and its elongation product, AdA, are incorporated into membrane phospholipids via acyl-CoA synthetase long-chain family member 4 (ACSL4) and lysophosphatidylcholine acyltransferase 3 (LPCAT3) catalyzed reactions. Through genome-wide screening approaches, ACSL4, LPCAT3, and cytochrome P450 reductase (POR) (Zou et al., 2020) have been identified as genes critical to ferroptosis. Phosphatidylethanolamine (PE)-linked arachidonic acid (PE-AA) and adrenic acid (PE-AdA) are considered the most vulnerable phospholipids to peroxidation. LOX-15 generates specific oxidation products like 15-hydroperoxy-AA-PEs or 15-hydroperoxy-AdA-PEs), which play pivotal roles in ferroptosis; moreover, (15-hydroperoxy)-di-acylated PE species acting as death signals (Kagan et al., 2017). High levels of 15-hydroperoxy-AA-PEs in cell membranes make them prone to oxidative cleavage, impairing functional proteins and cellular integrity (Ren et al., 2020; Ou et al., 2022). Additionally, 15-hydroxyeicosatetraenoic acid, the major 15-LOXs metabolite of AA, induces endothelial tight junction disruption and blood-brain barrier (BBB) dysfunction (see review on BBB in ferroptosis and references therein (Chen X. et al., 2022)**.**

**FIGURE 2:**
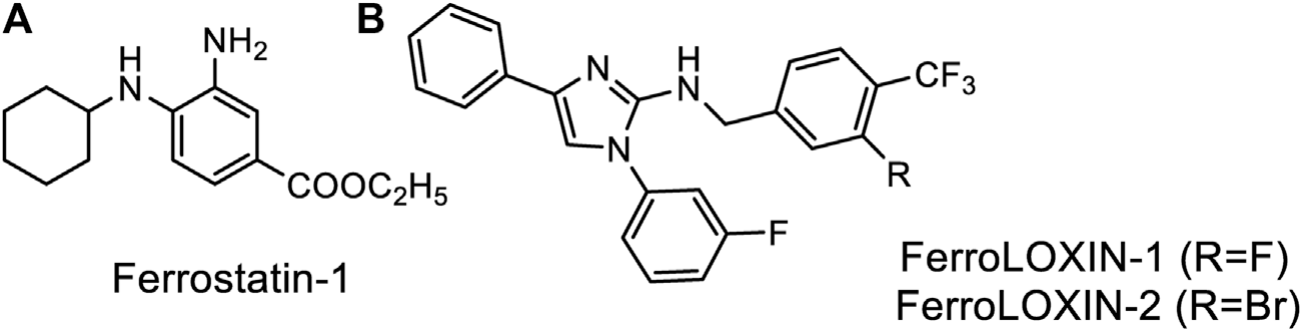
Specific inhibitors of Ferroptosis. **(A)** Ferrostatin-1, **(B)** newly developed FerroLOXINs targeting the complex between LOX15 and PEBP1 and inhibiting LOX15 activity.

Classification of the experimentally observed cell death as ferroptosis is primarily based on its inhibition by a small molecule inhibitor, ferrostatin-1 (Fer-1) (Figure 2A), that supposedly works as a radical trapping antioxidant. The inhibition of LOX-catalyzed peroxidation with Fer-1 has not been observed, thus supporting Fer-1 action on non-enzymatic peroxidation. However, a modest inhibition (ca. 2-fold) has been reported for the catalytic activity of a complex formed by LOX-15 and phosphatidylethanolamine-binding protein 1 (PEBP1) (Anthonymuthu et al., 2021). The design of inhibitors explicitly targeting the enzyme complex yielded two lead compounds, FerroLOXIN-1 and 2 (Figure 2B), which effectively suppressed ferroptosis *in vitro* and *in vivo* without radical scavenging or iron chelation. The inhibition by FerroLOXINs occurs via a specific interaction with the LOX-PEBP1 complex: they may change the substrate binding mode to the complex or block oxygen from reaching the catalytic center, preventing the peroxidation process from happening (Dar et al., 2023).

The debate about whether specific or non-specific peroxidation plays a role in ferroptosis continues because not all substances that scavenge radicals can stop ferroptosis. For example, the concentration of Ferrostatin-1 needed to stop ferroptosis is in a low micromolar range, whereas non-specific radical scavengers like N-acetyl cysteine or lipoic acid work in a non-physiological sub-millimolar to millimolar range (Liu Y. et al., 2020). It is becoming increasingly clear that the time of inhibitor administration matters for the outcome, but, unfortunately, no detailed time-course investigation of ferroptosis inhibition with any inhibitor was published to let us judge the actual time window of inhibitors, their protective effects, and their actual targets in the ferroptosis pathway.

### 2.3 The role of mitochondria in ferroptosis

The role of mitochondria in ferroptosis is still under debate since GPX4 inhibition-induced ferroptosis occurs in the cancer cell line with depleted mitochondria (Tait et al., 2013; Gao et al., 2019). However, the same study showed that mitochondrial depletion inhibits ferroptosis induced by erastin (Gao et al., 2019), meaning that mitochondria play a significant role in cysteine deprivation/glutathione depletion-induced cell death. Blocking the system Xc-, which leads to glutathione depletion, does not always trigger ferroptosis in cancer cells. Erastin, under specific conditions of low concentration (Huo et al., 2016; Sun et al., 2020) incubation time, and the level of VDAC1 expression (Huo et al., 2016), may cause apoptosis in cancer cells, but in developing neurons, it does cause non-apoptotic cell death. The glutathione depletion-induced cell death known for a while as “oxytosis” has been widely used to model oxidative stress in neurodegeneration; however, it is now classified as ferroptosis (Tang and Kang, 2023). Researchers are trying to understand how glutathione depletion, mitochondrial iron, and imbalance in ROS are connected. Recent studies suggest that blocking the cystine transporter leads to a buildup of glutamate, which undergoes the glutamine-glutamate-αKetoglutarate (αKG) conversion in the mitochondria to feed the TCA cycle. This process, called glutaminolysis, combined with cysteine/cystine starvation, exacerbates oxidative stress and disrupts iron balance (Gao et al., 2019). In several reports, exposure to glutamate, RSL3, and erastin was reported to cause a profound loss of mitochondrial membrane potential (Neitemeier et al., 2017)**.** However, there is no consensus on ferroptosis induction due to changes in mitochondrial membrane potential.

**FIGURE 3:**
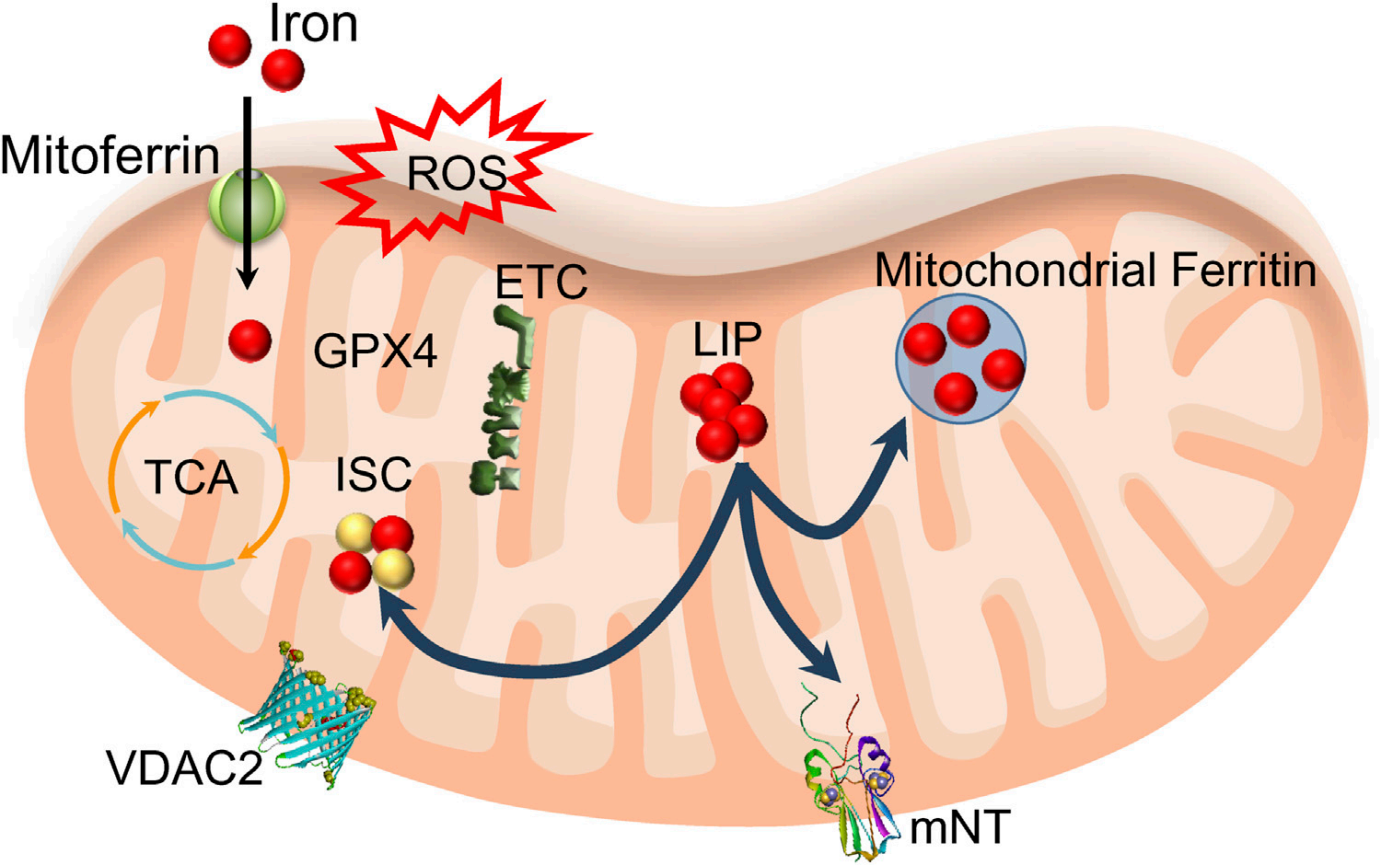
Mitochondria’s role in utilizing and storing iron. Iron enters mitochondria through mitoferrin and is used to synthesize heme (not shown) and iron-sulfur clusters (ISC). Mitochondrial LIP feeds ISC and mitochondrial ferritin. ROS produced from oxidative phosphorylation (ETC) may cause lipid peroxidation if the activity of mitochondrial GPX4 is compromised. Export of ISC occurs with the help of mitoNEET proteins (mNT). This process likely includes VDAC2/3 participation.

Mitochondria play a crucial role in utilizing and storing iron. Ferrous iron from the cytoplasm can enter the inner mitochondrial membrane (IMM) through a pathway driven by the membrane’s electrical potential or assisted by ferritin, crossing via mitoferrin one or mitoferrin 2 (Figure 3). Once inside, this iron is primarily used for the synthesis of heme and iron-sulfur clusters (ISC), as well as for storage within the mitochondria. Mitochondrial ferritin is a specific protein dedicated to storing iron within mitochondria, contributing to the establishment of mitochondrial iron pools and ensuring iron homeostasis (Sun et al., 2022). The observation that overexpression of mitochondrial ferritin protects the SH-SY5Y neuroblastoma cells from erastin-induced ferroptosis supports the hypothesis that imbalance in iron homeostasis inside mitochondria is induced by erastin treatment (Wang et al., 2016). Heme synthesis begins in the mitochondria and then continues in the cytosol, with the final insertion of iron into the porphyrin taking place in the mitochondria. However, heme synthesis does not seem to play a role in ferroptosis. The role of heme oxygenase-1, an enzyme that breaks down heme, in the initiation and propagation of ferroptosis is still uncertain (see below section 4.4). However, malfunction in iron-sulfur cluster synthesis and removal from mitochondria likely has a particular significance for the iron imbalance in mitochondria observed during ferroptosis, as discussed below.

### 2.4 VDAC2 as a potential link to iron imbalance

A class of porin channels, namely, voltage-dependent anion channels (VDACs) or mitochondrial porins, are the gatekeepers in the outer membrane of mitochondria (OMM), guiding the flow of mitochondrial metabolites in and out, thereby controlling the crosstalk between the mitochondria and the rest of the cell during oxidative stress (Tang D. et al., 2021). It has been observed that erastin targets VDAC2 specifically, and without VDAC2, ferroptosis does not happen (Xie et al., 2016). After being treated with erastin for 10 h, both VDAC2 and VDAC3 are no longer detectable because erastin activates an enzyme called NEDD4 E3 ubiquitin ligase, which clears VDAC2 and VDAC3 channels, making the cells resistant to erastin (Yang et al., 2020). What is so special about VDAC2 out of the three VDACs that consider this specific isoform a direct player in the ferroptosis mechanism?

Humans encode three VDACs: for VDAC1, the crystal structure has been resolved; for VDAC2, the NMR structure data were collected, and it was concluded that its structure closely resembles that of Zebrafish VDAC2 (Eddy et al., 2019). VDACs are established cell death targets in PD and regulate apoptosis (He et al., 2022). Glutamate interacts with VDAC1 *in vitro* (Gincel and Shoshan-Barmatz, 2004) and, as recently demonstrated, causes mitochondrial fragmentation through its effect on VDAC oligomerization in a model of glutamate-induced glutathione depletion (Nagakannan et al., 2019). VDAC2 is unique in possessing nine cysteine residues that act like sensors on the inner side of OMM, detecting oxidative stress and changes in redox status (Supplementary Figure S1). When cells experience oxidative stress or glutathione depletion, VDAC2 can be chemically modified: it can be carbonylated by lipid-derived molecules (a novel cysteine site has been discovered) (Chen et al., 2018), glutathionylated (Reina et al., 2020), or phosphorylated by GSK3β kinase (activated by oxidative stress and translocated to mitochondria) (Tanno et al., 2014). GSK3β is considered a significant positive regulator of the mitochondrial permeability transition pore, whereas VDAC2 is an anti-apoptotic modulator (Heslop et al., 2021). Malonylation of VDAC2 at lysine 46 is a marker of mitochondria-related ferroptosis (She et al., 2023). Additionally, VDAC2 is implicated in autophagy through interaction with Beclin 1 (Kang et al., 2018). It is now agreed that VDAC2 is more of a sensor of mitochondrial ROS rather than an ion transporter (Naghdi and Hajnoczky, 2016). However, how VDAC2’s sensing ability is strictly connected to iron imbalance in the cell and mitochondria is still unclear.

Concerning recent publications on VDAC1 (Lipper et al., 2019) and VDAC2/3 interactions (Wan et al., 2015) with mitoNEET (encoded by CDIS1 gene) and direct evidence of the mitoNEET’s role in ferroptosis (Yuan et al., 2016), we hypothesize that the disruption in the mechanism of iron-sulfur cluster transfer from mitochondria could be linked to the iron homeostasis imbalance and subsequent accumulation of labile iron to drive ferroptosis. NEETs have emerged as new targets in ferroptosis (see subsection 4.3). Overall, understanding the role of VDAC2 and its interactions with other molecules could provide insights into the mechanisms of ferroptosis and iron homeostasis. However, many unanswered questions remain, including whether erastin directly interacts with VDAC2.

### 2.5 Iron metabolism and ferritinophagy in ferroptosis

Iron is the most essential microelement, and iron-containing proteins contribute 5% towards all proteins in the organism. Iron plays a crucial role in numerous physiological functions within the brain, such as oxygen transport, mitochondrial respiration, DNA synthesis, and the synthesis and breakdown of neurotransmitters (Cao and Dixon, 2016; Jakaria et al., 2021). There are specific systems at the blood-brain barrier (BBB) and the blood-cerebrospinal fluid barrier to control how iron is distributed in the brain. Iron can enter the brain in two ways: transferrin receptor-mediated endocytosis and other transport systems for non-protein-bound iron (Viktorinova and Durfinova, 2021; Xu et al., 2023). For iron uptake, iron in the bloodstream binds to transferrin (TF), forming a complex with the transferrin receptor (TfR) on cell membranes, and is brought inside the cell through a process called endocytosis. Within the endosome, the six-transmembrane epithelial antigen of prostate 3 (STEAP3) assists in converting Fe3+ (ferric iron) to Fe2+ (ferrous iron), which is then released into the cell cytoplasm through divalent metal transporter 1 (DMT1) (Chen et al., 2020). The role of iron in ferroptosis has been extensively reviewed (Chen et al., 2020). While it is unclear whether the Fenton reaction or iron-containing enzymes are more critical in ferroptosis, an enzymatic reaction likely triggers the process. The LIP in cells, which includes iron not bound to proteins, is thought to play a crucial role in propagating the reactions that lead to lipid peroxidation, a hallmark of ferroptosis.

**FIGURE 4:**
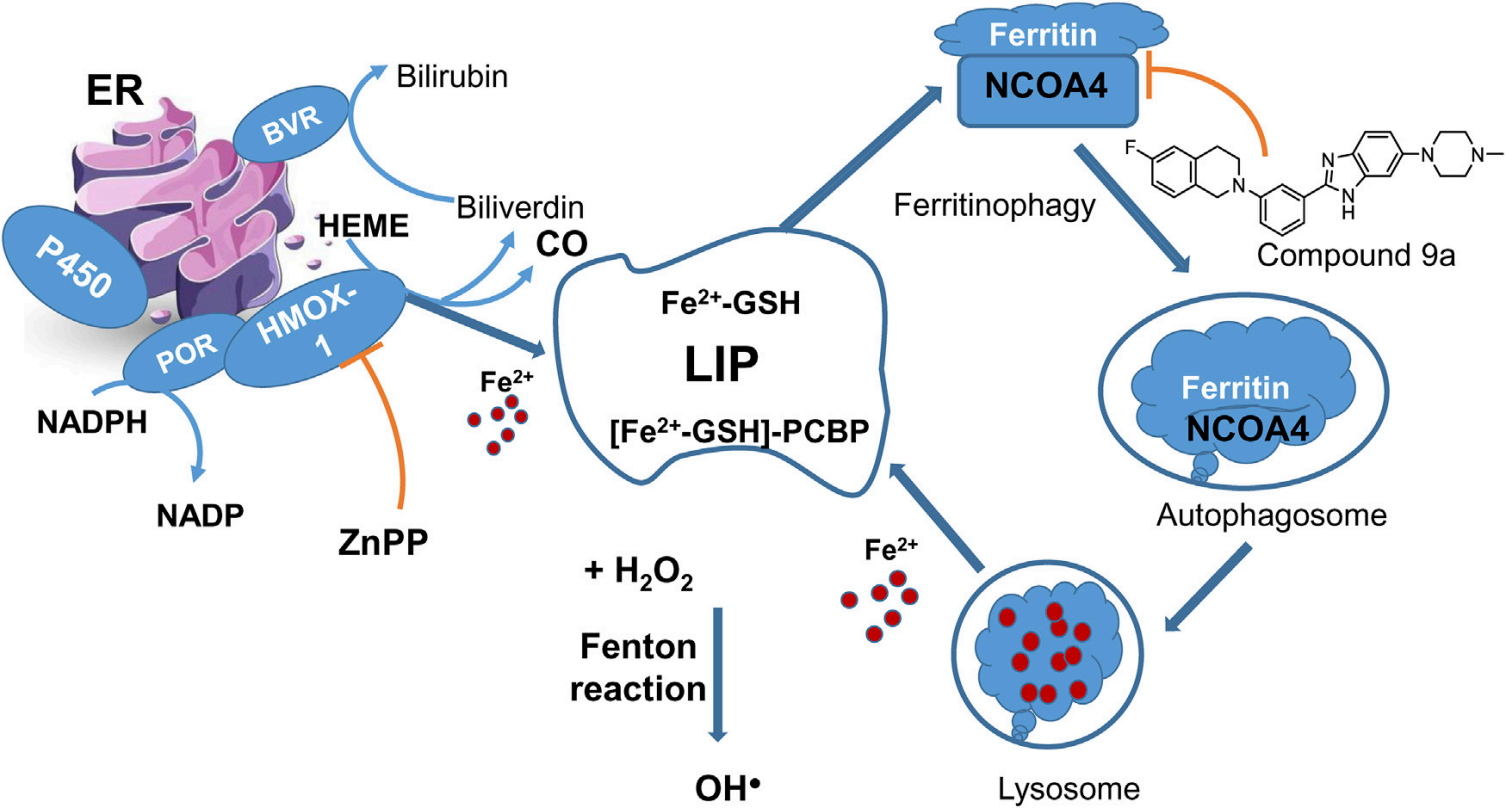
LIP maintenance and ferritinophagy. HMOX-1 in complex with POR is one of the sources of ferrous ions. HMOX-1 inhibition with ZnPP counteracts ferroptosis. Ferritinophagy presents a cycle returning iron to LIP. A new ferroptosis inhibitor **9a** targets the nuclear receptor coactivator 4 (NCOA4), a cargo receptor for ferritinophagy, and thus prevents the formation of ferritin-NCOA4 complex and subsequent ferritin degradation to feed the labile iron pool.

LIP describes the chemically reactive, kinetically exchangeable pool of iron used for iron cofactor synthesis, assembly, and insertion. The empirical evidence indicates that the LIP comprises iron in the ferrous state, coordinated by a polydisperse buffer system consisting of micro- and macro-molecules (Philpott et al., 2020). Cytosolic ferrous ion is coordinated by GSH forming the cytosolic LIP, with >90% of which is coordinated by iron chaperones, PCBPs that bind GSH coordinated ferrous ion with high affinity in a 1:1 ratio (Figure 4). PCBP1 and PCBP2 are the major iron chaperones that play integral roles in intracellular iron trafficking (see also Section 2.1 on LOX-15 translational repression by PCBP1). Unchaperoned free ferrous iron may react with hydrogen peroxide to generate hydroxyl radicals (Fenton reaction). While it is unclear whether the Fenton reaction or iron-containing enzymes are more critical in ferroptosis, an enzymatic reaction likely triggers the process.

Ferritin is an essential protein in the cytoplasm and the primary storage for ferrous ions (Reichert et al., 2020) through interaction with PCBPs. Ferritin is composed of ferritin light chain (FTL) and ferritin heavy chain 1 (FTH1), playing distinct roles in managing iron: FTH1 primarily converts Fe2+ to Fe3+, while FTL facilitates the entry of the next Fe2+ into the ferroxidase site (Yan HF. et al., 2021). Consequently, the amount of ferritin in a cell affects its sensitivity to ferroptosis: higher levels of ferritin means more iron is stored as Fe3+, making cells more resistant to ferroptosis because there’s less iron in the labile iron pool (Masaldan et al., 2019; Stockwell et al., 2020).

Accumulated evidence points to a link between autophagy and ferroptosis at the molecular level, as discussed in (Lee et al., 2023). A new autophagy process linked to ferroptosis is called ferritinophagy, which involves the selective removal of ferritin by a protein called nuclear receptor coactivator 4 (NCOA4). In ferritinophagy, NCOA4 recognizes ferritin and helps deliver it to the autophagosome, within which the ferritin undergoes degradation, releasing iron in the lysosome (Figure 4). When ferritinophagy is blocked by stopping autophagy or reducing the levels of NCOA4, the build-up of labile iron and ROS is prevented, ultimately preventing ferroptosis (Gao et al., 2016). NCOA4 has been identified as a potential target for developing drugs that inhibit ferroptosis by interfering with its interaction with ferritin (Figure 4). A new ferroptosis inhibitor, labeled 9a (IC50 of 300 nM), has been developed and tested in various models, showing promising results in reducing ferroptosis (Fang et al., 2021). It was about an order of magnitude less potent than ferrostatin-1 (IC50 = 40 nM) but much more effective than deferoxamine (IC50 = 3,860 nM) under the same experimental conditions in the cell models of erastin or RSL3-induced ferroptosis. In a rat model of ischemic stroke, compound 9a significantly reduced injury caused by lack of blood flow (ischemic reperfusion). This compound works by disrupting the interaction between NCOA4 and FTH1, a component of ferritin, thereby reducing the amount of available intracellular ferrous iron. Notably, NCOA4 is increased in low-oxygen conditions (hypoxia) and is regulated by hypoxia-inducible transcription factor HIF (Li et al., 2020a). However, there are some conflicting findings regarding the role of ferritinophagy in ferroptosis induced by erastin and RSL3. While compound 9a effectively inhibits ferroptosis (Fang et al., 2021), triggered by these ferroptosis inducers, a previous study suggested that ferritinophagy is elevated after erastin treatment but not influenced by RSL3 (Gryzik et al., 2021). In our viewpoint, ferritinophagy may not be the starting point of ferroptosis, but it is the final step before the process branches via LIP, and at this stage, the process can still be interrupted or stopped.

A process that connects mitochondrial iron and iron regulation in cells involves the transfer of iron-sulfur clusters (ISC) from mitochondria to iron regulatory protein 1 (IRP1) through mitoNEET and VDAC (Tam and Sweeney, 2023). Under normal conditions, IRP1 exists in its apo form with iron response element (IRE) binding ability. IRP1 binding to IRE within the 5′UTR results in translational repression (ferritin and ferroportin), whereas binding to IRE within the 3′UTR confers mRNA stability from RNase degradation (transferrin receptor). Under high iron resulting in oxidative stress, such as in pathological conditions, mitoNEET can transfer its ISC to IRP1, which loses its IRE binding ability. As a result, the translation of ferritin and ferroportin is upregulated due to a loss of IRP1 repression, whereas the transferrin receptor is downregulated due to a loss of mRNA stability.

IRP2 was identified as a critical ferroptosis regulatory gene identified using a high-throughput shRNA screening in the seminal work by Dixon *et al* (Dixon et al., 2012). Unlike IRP1, IRP2 lacks ISC and is primarily regulated by alterations in protein stability, which is sensitive to hypoxia and heme-induced oxidation. However, recent studies have shown that IRP2 can regulate iron levels and sensitivity to ferroptosis independently of IRP1 by sensing ISC cluster deficiency (Terzi et al., 2021). When the assembly of ISC proteins in the cytosol is inhibited, IRP2 stability and mRNA binding activity are increased. This suggests that triggering ferroptosis by restricting cysteine availability and thus limiting sulfur (from cysteine) for ISC synthesis would also mimic a response to iron starvation by increasing the mRNA binding activity of IRP1 and IRP2. However, the mRNA binding activity of neither IRP1 nor IRP2 has been characterized following the treatment with traditional ferroptosis-inducing agents like erastin or RSL3. Notably, in the context of brain function, IRP2 plays a pivotal role in regulating iron levels. Dysregulation of IRP2 is closely linked to the accumulation of iron observed in neurodegenerative conditions (Zhou et al., 2020).

## 3 Ferroptosis in neurodegenerative diseases

### 3.1 Neuroinflammation and ferroptosis

Neuroinflammation is a common etiopathogenic feature in various neurodegenerative diseases, including Alzheimer’s disease (AD), Huntington’s disease (HD), Parkinson’s disease (PD), stroke, and amyotrophic lateral sclerosis (ALS). It eventually leads to different types of regulated cell death, such as apoptosis, necroptosis, pyroptosis, and ferroptosis (Moujalled et al., 2021). Disruption of iron homeostasis is associated with neuroinflammation, indicating its role in ferroptosis. The activation of inflammatory signaling pathways is closely related to the occurrence of ferroptosis, and *vice versa* (Chen et al., 2023a): a) activated Janus kinase signal transducer and activator of transcription (JAK-STAT) pathway results in activated STAT1, which inhibits system xC-; b) NF-kB signaling is known to promote ferroptosis in tumors and inhibit Nrf2 pathway (Gao et al., 2021), however, through TNF-α, it activates transcription of miR-155, which binds Bach1 mRNA and hinders its translation, and thus activates HMOX-1 expression (Pulkkinen et al., 2011); c) in cGAS-STING pathway, STING, an essential element in the type I interferon response and verified inducer of ferroptosis, was recently shown to interact with NCOA4 to exacerbate ferritinophagy (Wu J. et al., 2022); d) in mitogen-activated protein kinase (MAPK) pathway, p38MAPK and extracellular-signal regulated kinase (ERK) mediate both inflammatory response and ferroptosis (Wang X. et al., 2023).

Deposition of aggregated proteins/peptides is a characteristic hallmark feature of chronic neurodegenerative diseases. Such aggregates can adsorb iron and acquire catalytic properties, producing ROS and promoting ferroptosis, among other types of cell death. Specific evidence for the role of ferroptosis in a particular disease setting is difficult to establish since neurodegenerative diseases exhibit multiple standard features, such as dysregulated iron homeostasis, oxidative stress, and diminished glutathione. Perhaps the best evidence comes from the protection afforded by specific ferroptosis inhibitors in preclinical disease models and, ultimately, their use in human clinical trials (see section 4.5).

### 3.2 Ferroptosis and Alzheimer’s disease

Several studies have highlighted a connection between ferroptosis and AD in the past decade (Ma et al., 2022). AD is characterized by chronic neuroinflammation, amyloid beta-protein deposition, and the hyperphosphorylation of tau protein. AD is considered a multifactorial disease with various etiologies and complicated pathophysiological processes. Previous research identified neuroinflammation and typical microglial activation as significant mechanisms underlying AD (Leng and Edison, 2021; Woodburn et al., 2021). Abnormally activated microglia release a panoply of pro-inflammatory factors that aggravate the dysregulation of iron homeostasis and neuroinflammation, forming a vicious cycle (Wang M. et al., 2023).

Both biochemical and morphological features of ferroptosis have been documented in postmortem brains of AD patients, including depletion of GSH, inactivation of GPX4, and imbalance in iron metabolism, resulting in lipid peroxidation, mitochondrial dysfunction, and ROS generation (Zhao et al., 2023). In AD, an accumulation of amyloid-beta (Aβ) and tau tangles may trigger ferroptosis by disrupting cellular iron balance and inducing oxidative stress (Jakaria et al., 2021). The involvement of iron in AD pathology is proven by the discovery of iron and ferritin around the glial cell in the senile plaques and neurofibrillary tangles. Synchrotron-based X-ray fluorescence microscopy detected abnormal iron enrichment in procured Aβ amyloid plaques from AD postmortem brain tissues (Huang, 2023). Iron interacts with amyloid-beta (Aβ) and tau by forming deposits and creating a peptide hemin complex *in vitro* (Pal and Dey, 2023). The Aβ- Fe^3+^ redox interaction mechanism has been validated in Aβ amyloid plaque cores from AD postmortem and transgenic mouse brain tissues (Huang, 2023). This interaction contributes to generating ROS, potentially playing a role in the ferroptotic cell death pathway (Lane et al., 2018).

An excessive build-up of iron in senile plaques and neurofibrillary tangles was found in various brain regions, particularly in the hippocampus. However, it remains uncertain whether this iron accumulation in these brain areas is linked to malfunctions in proteins regulating either the entry of iron into cells through DMT1 (an iron importer) or the removal of iron from cells via ferroportin (an iron exporter) (Crespo et al., 2014). An upregulation in ferritin FTH and FTL expression has been noted in AD brains, indicating a potential increase in LIP levels. Additionally, compensatory ceruloplasmin elevation is observed in AD, aiding in oxidizing Fe2+ to Fe3+ to facilitate FPN-mediated iron export. However, abnormal downregulation of FPN levels in AD reduces iron export, resulting in elevated LIPs within cells and triggering lipid peroxidation, a marker of ferroptosis (Ashraf et al., 2020; Zhou et al., 2020).

The amyloid precursor protein (APP) regulates neuronal iron content by promoting iron export through stabilizing FPN. On the other hand, iron is an inductor for furin, which reduces the activation of α-secretase, which leads to the activation of β-secretase and contributes to amyloid plaque production (Hwang et al., 2006; Altamura and Muckenthaler, 2009). Tau, a microtubule-associated protein, assists in transporting APP to the cell membrane and stabilizing the FPN1-APP complex. Therefore, tau inactivation affects APP transport to the cell membrane, leading to iron accumulation and oxidative stress-induced cell death, potentially linked to ferroptosis in AD (Belaidi et al., 2018; Dlouhy et al., 2019; Zhou et al., 2020). In both human AD and APP-transgenic mice brains, notably decreased levels of hepcidin and ferroportin were observed in hippocampal lysates (Raha et al., 2013).

Ayton *et al* suggested that iron levels in the brain could predict AD progression, especially in individuals carrying the apolipoprotein E (APOE) gene, notably the APOE-e4 risk allele. Research on APOE4 carriers suggests that this genetic variant may induce iron deposition in AD brains by reducing the delivery of high-density lipoprotein, a significant regulator of intracellular iron levels. Ayton’s results have revealed that ferritin is closely associated with the level of apolipoprotein E, and the APOE-e4 allele elevates the risk of AD (Ayton et al., 2015).

A large body of research has shown extensive mitochondrial abnormalities in AD (Wang et al., 2020). Mitochondria play multiple regulatory roles in ferroptosis, and preserving mitochondrial integrity is considered an effective strategy to prevent ferroptosis (Wu et al., 2021). A recent study by Li *et al* demonstrated an intriguing connection between mitophagy-dependent ferroptosis through the CD36/PINK1/Parkin pathway leading to blood-brain barrier (BBB) destruction in AD (Li et al., 2022). They showed that Aβ1-40 aggravated BBB damage by binding to CD36, a phagocytic receptor in the pericytes, a vital component of the neurovascular unit and the BBB. They observed that Aβ1-40 accumulation resulted in ferroptosis accompanied by a rise in Fe2+, increased lipid ROS production, decreased GSH-Px, and inhibited GPx4 and XcT in the pericytes. Interestingly, Aβ1-40 caused autophagy-dependent ferroptosis mediated through the PINK1/Parkin pathway, which was blocked by Mdiv-1, a putative inhibitor of mitochondrial fission protein dynamin-related protein 1 (Drp1), which causes excessive mitochondrial fragmentation. However, genetic deletion of CD36 expression alleviated Aβ1-40 mediated increased BBB permeability and autophagy-dependent ferroptosis. These data suggest a pathogenic role of mitochondrial autophagy through the CD36/PINK1/Parkin pathway in ferroptosis in AD.

To summarize, disturbances in iron balance, iron regulatory proteins, lipid peroxidation, and defective mitochondrial autophagy collectively suggest potential implications of ferroptosis in AD.

### 3.3 Ferroptosis and Parkinson’s disease

Parkinson’s disease (PD) is a common late-onset progressive neurodegenerative disorder characterized by loss of dopaminergic (DA) neurons in the substantia nigra pars compacta (SNpc) and the presence of fibrillar cytoplasmic inclusions composed of alpha-synuclein (αS) called Lewy Bodies (Thomas and Beal, 2007), as well as iron deposition (Altshuler and Hyde, 1985). Although the pathological mechanisms underlying PD are not fully understood, the generation of lipid peroxides, defects in the antioxidant system, and iron dysregulation are implicated in ferroptosis. A recent study exploring the DNA methylation patterns in a cohort of 1,132 individuals with PD and 999 controls identified a correlation between increased methylation in the promoter region of the SLC7A11 gene, responsible for encoding the cysteine-glutamate antiporter system Xc-, and the risk of PD. This aberrant hypermethylation in SLC7A11 reduces system Xc-expression, potentially causing the decline in intracellular glutathione (GSH) levels in PD cases and potentially augmenting susceptibility to ferroptosis (Vallerga et al., 2020).

The DA neurons are particularly susceptible to PD, and ferroptosis is implicated in their selective vulnerability due to iron-induced oxidative stress. Chronic inflammation in the SN accompanies conditions with progressive neurodegeneration by gradually increasing iron deposition (Thomsen et al., 2015). Microglial iron accumulation is involved in the occurrence and development of PD, and inhibition of microglial iron deposition prevents neuroinflammation (Liu et al., 2022). The SNpc and brainstem of PD patients have iron deposition, and the severity of the disease is related directly to the iron concentration (Mahoney-Sanchez et al., 2021; Lin et al., 2022). Consistent with these findings, a study that combined MRI imaging, quantitative susceptibility mapping, and regional gene profiling in a cohort of PD patients and control subjects showed a significant increase in iron accumulation in PD compared to controls. Genes related to heavy metal detoxification were differentially expressed in distinct brain cell types of PD patients, suggesting regional and selective vulnerability to iron accumulation in PD (Thomas et al., 2021). Elevated levels of DMT1, observed in the SNpc of PD patients and various PD mouse models, likely contribute to increased cellular iron intake (Salazar et al., 2008; Bi et al., 2020). Moreover, reduced levels of ferritin in the SN region have been reported in post-mortem brains of individuals with PD (Bi et al., 2020). Although this evidence suggests a significant role of iron imbalance and ferroptosis in mediating neurodegeneration in PD, how these contribute to each other and lead to neurodegeneration is still elusive. Nevertheless, given the vital link between iron accumulation and PD pathogenesis, iron chelation therapy offers the chance to provide a critical change in the PD treatment paradigm. However, a randomized study of iron chelation with deferiprone (FAIRPARK-II trial) without any DA replacement treatments unexpectedly showed worse outcomes for patients with PD (Devos et al., 2022). These results warrant further research into the biological process of iron accumulation and associated neurodegenerative mechanisms in PD pathogenesis.

Besides being a major component of Lewy body pathology, both genetic and biochemical abnormalities of αS are associated with PD pathogenesis. However, neither αS’s physiological function nor neuropathological mechanisms are entirely understood to date. It has been shown that αS binds to iron in Fe2+ and Fe3+ states, with the Fe3+ form being responsible for enhanced αS fibril formation and aggregation (Uversky et al., 2001). Iron accumulates in the SN of the midbrain, and postmortem analysis from PD patients shows the colocalization of iron and αS in Lewy bodies (Castellani et al., 2000). Further, αS mRNA contains an iron response element (IRE) within its 5′UTR, indicating that its translation is responsive to iron metabolism (Chen et al., 2019). Iron accumulation also results in lysosomal dysfunction, an organelle responsible for the clearance of αS aggregates, by inhibiting autophagosome-lysosome fusion and the activity of lysosomal hydrolases (Wildburger et al., 2020). These findings indicate a crucial connection between iron metabolism and αS aggregation in PD pathogenesis.

αS is also known to determine the sensitivity of DA neurons to ferroptosis by generating lipid peroxides. A recent report by Angelova *et al* showed that exogenous αS oligomers bind to the plasma membrane to drive ferroptosis by generating lipid peroxides, which can be rescued by iron chelators, D-PUFAs, or ferrostatin-1 (Angelova et al., 2020). The significance of lipids in PD is further highlighted by data showing the impact of αS on lipid metabolism, modulation of αS by lipids, and the identification of genetic determinants involved in lipid homeostasis associated with synucleinopathies (Alza et al., 2019). Consistent with these findings, endogenous αS plays a pivotal role in the survival of DA neurons by regulating phospholipid membrane composition, specifically, the ether-linked phospholipids essential for ferroptosis. Reduction of αS expression in human DA neuronal models markedly decreased the proportion of ether-phospholipids in the plasma membrane to a comparable level as when the primary ferroptotic regulator ACSL4 was reduced. Conversely, elevated levels of αS expression rendered DA neurons more vulnerable to ferroptosis-induced lipid peroxidation and cell death (Mahoney-Sanchez et al., 2022). These results directly link an established lipid peroxidation pathway essential for ferroptosis and the implicated role of αS in lipid metabolism. However, downstream pathways whereby lipid peroxidation leads to DA neuronal dysfunction and death are not fully understood.

In summary, a significant correlation exists between ferroptosis and PD pathophysiological features, such as abnormal iron metabolism, increased lipid peroxidation, decline in intracellular GSH, and downregulation of the cystine-glutamate antiporter system (Xc-), and αS aggregation. Further understanding the intricate relationship between these pathophysiological processes driving PD pathophysiology may pave the way for a better understanding of ferroptosis in PD.

## 4 Protective pathways and novel therapeutic targets against ferroptosis

### 4.1 GPX4-independent protective pathways

**FIGURE 5:**
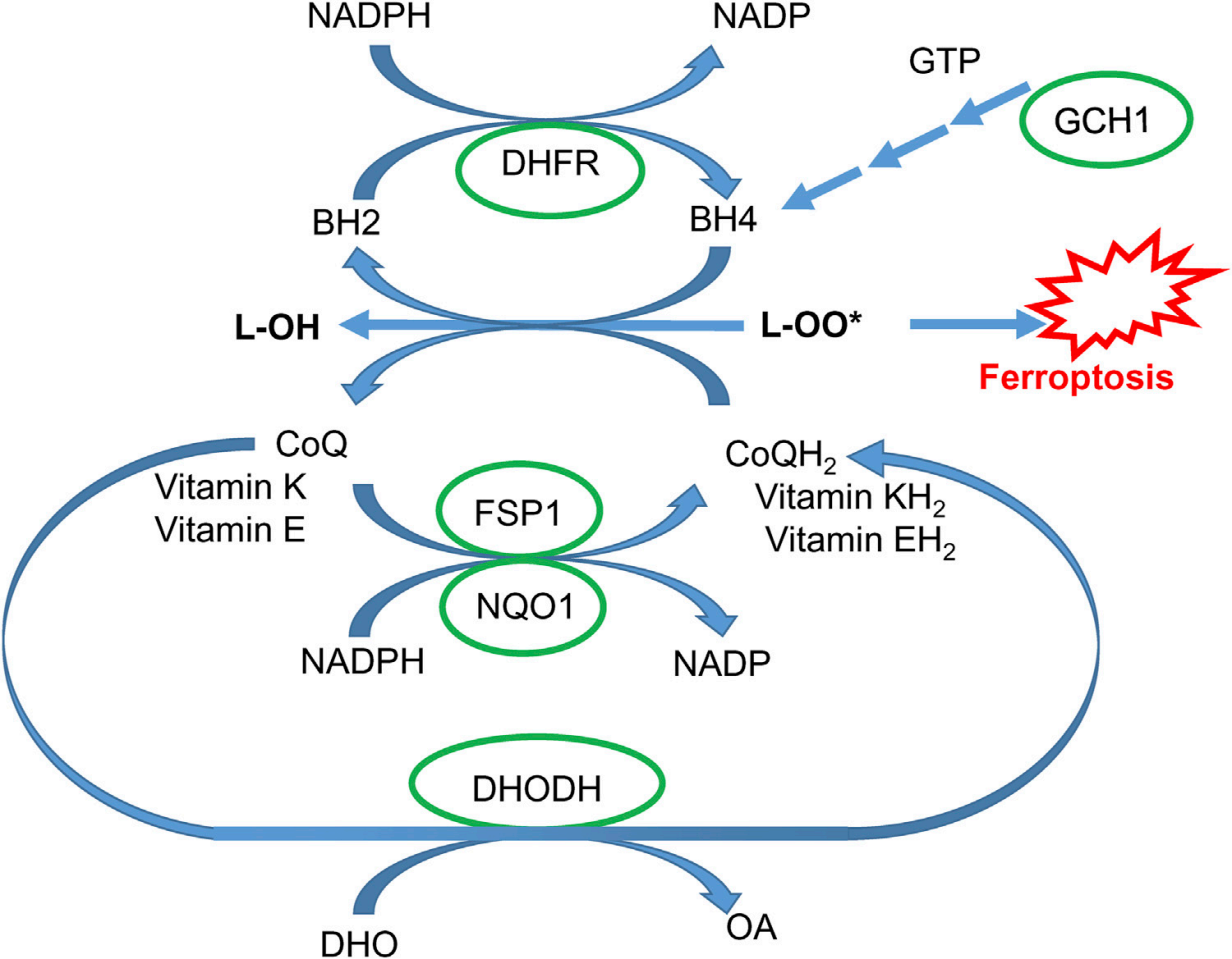
GPX4-independent protective pathways. Coenzyme Q10 in reduced form (CoQH_2_) and vitamins K and E reduce lipid peroxides and are regenerated via NADPH-dependent FSP1- and NQO-1-catalyzed reaction. Coenzyme Q10 can also be regenerated via a DHODH-catalyzed reaction (DHO, dihydroorotic acid; OA, orotic acid). Tetrahydrobiopterin BH4 can effectively quench lipid peroxy-radicals and is regenerated via a dihydrofolate reductase (DHFR)-catalyzed reaction. BH4 is synthesized via a sequential enzymatic reaction, with the GCH1 enzyme being rate-limiting (see the text for details).

In addition to the GPX4-dependent system, ferroptosis is also regulated by three other systems related to oxidation-reduction processes (Liu et al., 2023). Two systems (Figure 5), NAD(P)H quinone oxidoreductase 1 (NQO1) and ferroptosis suppressor protein 1 (FSP1), catalyze the reduction of coenzyme Q10 (CoQ_10_) (Bersuker et al., 2019; Doll et al., 2019) to work alongside the glutathione-GPX4 pathway and prevent ferroptosis. FSP1, previously known as apoptosis-inducing factor mitochondrial 2 (AIFM2, also recognized as AMID or PRG3), does not promote cell death (Novo et al., 2021) but functions as a NAD(P)H-dependent flavo-oxidoreductase. It protects cells from ferroptosis by catalyzing the reduction of CoQ_10_, vitamin K, and α-tocopheryl radical, which terminate the chain reaction of lipid peroxidation by neutralizing harmful lipid peroxy- and oxyradicals (Mishima et al., 2022; Li et al., 2023). Recent research has identified FSP1 as a gene targeted by nuclear factor erythroid-derived 2-related factor 2 (Nrf2) (Mishima et al., 2022)**.** Another protective pathway is associated with the catalytic activity of dihydroorotate dehydrogenase (DHODH), which defends against both hypoxia (Spinelli et al., 2021) and ferroptosis (Mao et al., 2021) by reducing CoQ_10_ (Figure 5).

The third protective system against ferroptosis is Guanosine triphosphate cyclohydrolase 1 (GCH1)-tetrahydrobiopterin (BH_4_) (Kraft et al., 2020). Mechanistically, BH4 acts as a reducer, neutralizing harmful lipid oxy- and peroxy-radicals. When BH4 is oxidized to BH2, it can be restored to its active form, BH4, through reduction by NADPH with the assistance of dihydrofolate dehydrogenase**.** Additionally, BH4 is a crucial cofactor for nitric oxide synthases (NOS). In BH4-deficient conditions, superoxide is generated instead of nitric oxide (NO) (Takimoto et al., 2005), contributing to ferroptosis. GCH1 is a rate-limiting enzyme driving BH4 synthesis. Mutations in GCH1 are associated with a higher risk of dopa-responsive dystonia and PD (Yoshino et al., 2018)**.**

Mammalian cells rely on two main pathways to synthesize GSH: importing cystine through system Xc- and synthesizing cysteine via the reverse transsulfuration pathway, the sole cysteine biosynthesis route. Cystathionine β-synthase (CBS), a gene regulated by Nrf2, catalyzes the condensation of homocysteine and serine to form cystathionine, which is then converted to cysteine by cystathione γ-lyase (CSE). Both the enzymes CBS (Liu N. et al., 2020) and CSE (Jamaluddin et al., 2022) are Nrf2 target genes. Disruption of the transsulfuration pathway is linked to several neurodegenerative diseases, including AD (Paul, 2021), PD (Corona-Trejo et al., 2023), and Huntington’s disease (Liu N. et al., 2020).

### 4.2 Inhibition of HIF prolyl hydroxylases (PHDs)

Iron chelators are thought to safeguard against ferroptosis by hindering lipid peroxidation fueled by the labile iron pool. However, it is tempting to speculate that iron chelators inhibit iron-dependent enzymes that contribute to ferroptosis initiation and progression. One of the potential candidates other than lipoxygenases could be αKG-dependent non-heme iron dioxygenases. These enzymes are essential in regulating the stability and properties of various transcription factors and in demethylating histones and DNA. Inhibition of these enzymes is observed in hypoxic conditions and when iron chelators are present. The effect of hypoxia on ferroptosis varies greatly depending on the context and cell type: hypoxia typically inhibits ferroptosis in cancer cells, whereas, in normal cells, it often triggers or enhances ferroptosis (Zheng et al., 2023). Hypoxia leads to the accumulation of HIF, which activates the expression of dozens of pro-survival genes, including those involved in iron regulation and protection against ferroptosis. HIF can be stabilized in the presence of iron chelators or inhibitors of HIF prolyl dehydrogenase (HIF PHD), which are αKG-dependent non-heme iron dioxygenase, such as adaptaquin (Karuppagounder et al., 2016). HIF PHDs exist in three forms and have multiple protein targets. A study showed that silencing PHD1, not PHD2 or PHD3, prevented ferroptotic death in HT22 cells and primary neurons (Siddiq et al., 2009). Transcriptomic analysis revealed that HIF PHD inhibition unexpectedly downregulated ATF4-dependent genes, including Trib3, Chac1, and Chop (Karuppagounder et al., 2016), all linked to ferroptosis. ATF4, like HIF, is a substrate for HIF prolyl hydroxylases, and mutant ATF4 lacking prolines, which are hydroxylated by the enzyme, blocked ferroptosis, suggesting a dominant-negative effect. Despite the established connection between ATF4 and ferroptosis, there is a lack of clear mechanistic justification for using HIF prolyl hydroxylase inhibitors to regulate the initiation and progression of ferroptosis. The commercial HIF PHD inhibitor, Roxadustat, has demonstrated contradictory effects: inducing ferroptosis in the glioblastoma (Su et al., 2022) while protecting against folic acid-induced kidney injury by reducing ferroptosis through Akt/GSK-3*β*-mediated Nrf2 activation (Li et al., 2020b). These conflicting findings underscore the need for further research on the involvement of HIF prolyl hydroxylase engagement in the ferroptosis pathway.

### 4.3 NEETs as new ferroptosis targets

There are three proteins (Mittler et al., 2019) with a CDGSH iron-sulfur domain encoded by CDIS genes where mitoNEET is encoded by CISD1, Miner one (or NAF-1) by CISD2, and Miner two (or miNT) by CISD3. Miner two is only found in mitochondria, exists as a monomeric unit, and can fit through VDAC1, a channel in the outer membrane of mitochondria, much like VDAC2 (shown in Supplementary Figure S1). MitoNEET and NAF-1 can exist as dimers in the outer mitochondrial membrane (OMM) and endoplasmic reticulum (ER), respectively. CISD1 was identified among ferroptosis-related genes involved in asthma (Wang H. et al., 2023), and psoriasis (Wu MN. et al., 2022). Inhibition of CISD1 reduced LPS-induced ferroptosis *in vivo* and *in vitro* by preventing mitochondrial membrane potential depolarization, cellular ATP reduction, and ROS accumulation (Zhang et al., 2024). CISD1-Dependent regulation of ferroptosis is shown in glutathione depletion–induced oxidative cardiomyopathy in murine hearts (Li et al., 2024). CISD1 expression has been recently demonstrated as a ferroptosis hub gene of prognostic relevance for triple-negative breast cancer (Yuan et al., 2023) and tumor immune filtration in gastric cancer (Zang et al., 2023). NAF-1 (CISD2) is localized to the outer membrane of mitochondria, ER membrane, and mitochondrion-associated ER membranes. NAF-1 is an NF-κB antagonist (Kung and Lin, 2021) and exerts anti-inflammatory effects (Lin, 2020)**.** NAF-1 deficiency causes accelerated aging and shortened lifespan (Chen et al., 2009), whereas persistent expression of CISD2 promotes longevity in mice (Wu et al., 2012).

#### 4.3.1 MitoNEET (CISD1)-a target in Parkinson’s disease

Mitochondrial dysfunction and toxic protein aggregates are crucial pathological features in the pathogenesis of PD. The E3 ligase Parkin and mitochondrial PTEN-induced kinase 1 (PINK1) are crucial for mitochondrial quality control: PINK1 phosphorylates and activates Parkin, which in turn ubiquitinates mitochondrial proteins, and helps maintain the mitochondrial homeostasis (Goncalves and Morais, 2021). The iron-sulfur cluster containing proteins MitoNEET and Miner two have been identified as substrates of Parkin in various proteomic studies. The *Drosophila* homolog of MitoNEET accumulates in Pink1 and Parkin mutant flies and during aging, and this accumulation is notably detrimental to neuronal survival (Martinez et al., 2024)**.** Loss of PINK1 and Parkin also leads to dysregulation of inositol 1,4,5-trisphosphate receptor (IP3R) activity, robustly increasing ER calcium release, and MitoNEET was shown to function downstream of Parkin to control IP3R directly (Ham et al., 2023)**.** On the other hand, mitochondria isolated from mice lacking mitoNEET had reduced capacity to produce ATP. Gait analysis revealed a shortened stride length and decreased rotarod performance in mitoNEET knockout mice, consistent with the loss of striatal dopamine (Geldenhuys et al., 2017)**.**

#### 4.3.2 MitoNEET (CISD1)-a target gene in ALS

Amyotrophic lateral sclerosis (ALS) is a neurodegenerative disease characterized by progressive muscle paralysis, which is followed by degeneration of motor neurons in the motor cortex of the brainstem and spinal cord. The etiology of sporadic ALS is still unknown, limiting the exploration of potential treatments. The autopsy and blood datasets from Gene Expression Omnibus were used to explore the role of ferroptosis and ferroptosis-related gene alterations in ALS. Gene set enrichment analysis (GSEA) found that the activated ferroptosis pathway displayed a higher enrichment score, and the expression of 26 ferroptosis genes showed noticeable group differences between ALS and controls. CISD1 was identified as one of the target genes through the least absolute shrinkage and selection operator analysis, in which gene signature could differentiate ALS patients from controls (Zhang et al., 2022).

#### 4.3.3 NAF-1 (CISD2)-a target in AD

CISD2 upregulation can ameliorate amyloid β (Aβ) toxicity and prevent neuronal loss using an AD mouse model (APP/PS1 double transgenic mice) (Wu et al., 2012)**.** A two-fold increase in CISD2 expression significantly promotes survival and alleviates the pathological defects associated with AD, whereas CISD2 deficiency accelerates AD pathogenesis. Overexpression of CISD2 protects against Aβ‐mediated mitochondrial damage, attenuates loss of neurons and neuronal progenitor cells, and shifts the expression profile of AD-dysregulated genes toward the patterns observed in wild‐type mice.

Pharmacological regulation of CISD2 expression by hesperitin slows aging and promotes longevity (Yeh et al., 2022). Of note, hesperitin, like many flavonoids, is a direct Nrf2 activator. CISD1 is a known Nrf2 target gene, and CISD2 is possibly too, since Nrf2 inhibition with its inhibitor ML385 blocks the neuroprotective effects of CISD upregulation in oxygen-glucose deprivation/reoxygenation model in HT22 cells (Hu et al., 2023).

### 4.4 Heme oxygenase–a promoter of ferroptosis?

Heme oxygenase-1 (HMOX-1) is an inducible enzyme known for its anti-inflammatory, antioxidant, and neuroprotective effects (Ryter, 2022). However, increased expression of HMOX1 during aging and age-related neurodegenerative diseases has been associated with neurotoxic ferric iron deposits. HMOX-1 is a primary intracellular source of iron and, thus, may play a pro-death role in ferroptosis via enhancing iron release (Ryter, 2021).

#### 4.4.1 Enzyme Complex with POR

HMOX-1 localizes to the ER membrane and catalyzes the reaction of heme degradation, yielding biliverdin, CO, and Fe2+. The ferrous iron released replenishes LIP, as shown in Figure 4. HMOX-1 forms a catalytic complex with cytochrome P450 reductase (POR), an enzyme providing electron transfer of two reducing equivalents from NADPH through enzyme-bound FAD and FMN to HMOX-1 (Muller-Enoch and Gruler, 2000). POR is anchored to the membrane and serves as a metabolic hub that activates specific P450 enzymes in response to chemical cues and local environment changes such as lipid composition and ionic strength. Using bioluminescence resonance energy transfer, POR-cytochrome CYP1A2 complex was shown to be readily disrupted by the addition of HMOX-1, but not the other way around (Connick et al., 2021). This indicates the POR-HMOX-1 complex remains stable against competitive disruption from cytochrome P450s. It has been demonstrated that HMOX-1 forms complexes with CYP1A2, CYP1A1, and CYP2D6, but not all P450 enzymes (Connick et al., 2021). Both HMOX-1 (Chen Q. et al., 2022; Luo et al., 2023) and POR (Zou et al., 2020) come out as ferroptosis hub-genes in multiple bioinformatics reports and are bonafide Nrf2/Bach1 target genes (Table. 1).

**TABLE 1:** Shared pathways modulated by Nrf2 and Bach1 bound ferroptosis and iron homeostasis genes.

Pathways	Gene name	Gene function
*Glutathione synthesis and Cellular redox homeostasis*	*Gclm*	*Glutamate-cysteine ligase regulatory subunit*
	*Gclc*	*Glutamate-Cysteine Ligase Catalytic Subunit*
	*Txnrd1*	*Thioredoxin reductase 1*, the predominantly cytosolic selenoprotein, is a key enzyme for protection against oxidative stress
	*Aox1*	*Aldehyde oxidase 1,* best known as drug metabolizing enzyme that catalyzes the hydroxylation of N-heterocycles and the oxidation of aldehydes to the corresponding acid
*Iron homeostasis, Iron-sulfur cluster enzymes, Heme metabolism*	*Fth1*	*The heavy subunit of ferritin, the* major intracellular iron storage protein
	*Abcb6*	*ATP Binding Cassette Subfamily C Member 6* or multidrug resistance-associated protein 6, an intracellular transporter associated with mitochondrial function, located in the mitochondrial-associated membrane, an organic anion efflux pump
	*Tet1*	*Ten-eleven translocation methylcytosine dioxygenase 1*, epigenetic reprogramming, involved in regulating iron homeostasis by demethylating the promoter of RNF217, the ubiquitin ligase responsible for the degradation of iron exporter ferroportin
	*Aco2*	*Mitochondrial aconitase 2* contains Fe_4_S_4_ ISC. The most sensitive TCA cycle enzyme to oxidative stress
	*Hmox1*	*Heme oxygenase 1* is responsible for cleaving heme to form biliverdin
*Arachidonic acid cascade*	*Tbxas1*	*Thromboxane A synthase 1 - a* member of the cytochrome P450 superfamily of enzymes - catalyzes the conversion of prostaglandin H_2_ to thromboxane A_2_, and to 12-Hydroxyheptadecatrienoic acid
*Adipogenesis and lipid metabolism*	*Por*	*Cytochrome P450 Oxidoreductase,* has a major role in the metabolism of drugs and steroids and can also provide electron transfer to heme oxygenase and cytochrome B5
	*Hmgcr*	*3-hydroxy-3-methylglutaryl-CoA reductase* is the rate-limiting enzyme for cholesterol synthesis
	*Agl*	*Amylo-Alpha-1,6-Glucosidase* - glycogen debrancher enzyme involved in glycogen degradation
*Protein degradation*	*Ubb*	*Ubiquitin b* targets cellular proteins for degradation by the 26S proteasome
	*Ubc*	*Polyubiquitin c* provides extra ubiquitin necessary to remove damaged/unfolded proteins
*Exo- and endocytosis*	*Cltc*	*Clathrin heavy chain* mediates intracellular trafficking of receptors and endocytosis of macromolecules
	*Zfyve28*	*Zinc Finger FYVE-Type Containing 28* is involved in insulin resistance by promoting phosphorylated insulin receptor degradation. Negative regulator of epidermal growth factor receptor (EGFR) signaling. Acts by promoting EGFR degradation in endosomes when not monoubiquitinated
	*Exoc6*	*Exocyst Complex Component 6* **-** Component of the exocyst complex involved in the docking of exocytic vesicles with fusion sites on the plasma membrane. Is Associated with the Risk of Type 2 Diabetes and Pancreatic β-Cell Dysfunction
	*Epb41*	*EPB41 erythrocyte membrane protein band 4.1* together with spectrin and actin, constitute the red cell membrane cytoskeletal network and play a critical role in erythrocyte shape and deformability
*Neurogenesis*	*Susd6*	*Sushi domain-containing protein 6,* a paralog of CMDS3, implicated in synaptogenesis and neurogenesis
*Transcription factors*	*Bach1*	*BTB domain and CNC homolog 1,* a transcription factor that inhibits Nrf2 transcription, and regulates cell survival pathways
	*Bach2*	*BTB domain and CNC homolog 2,* is a transcription factor which mediates variety of immunomodulatory functions
	*Nrf2*	*Nuclear factor erythroid 2-related factor 2, is a transcription factor,* master regulator of cellular stress response, facilitating the expression of cytoprotective genes, including those responsible in immunomodulation, and iron metabolism

#### 4.4.2 Is POR a producer of hydrogen peroxide?

The POR quantity in the cell is limited; in other words, POR typically exists only within the protein complexes with an electron acceptor enzyme in the ER membrane. However, forced overexpression of POR generates a free, unbound enzyme, which, like any NAD(P)H-dependent dehydrogenase, in the absence of electron acceptor substrate, may catalyze a side reaction of oxygen reduction to hydrogen peroxide using NADPH reducing equivalents (Yan B. et al., 2021). Then, if available, free ferrous ions may react with hydrogen peroxide to initiate the Fenton reaction. Whether there is a mechanistic link resulting in POR overexpression upon ferroptosis induction is still unclear.

In general, ER is a compartment with more pro-oxidative balance to support protein folding, maintaining a reduced glutathione/oxidized glutathione (GSH:GSSG) ratio closer to 3:1 (Dixon et al., 2008). The folding process relies on protein disulfide isomerase working in concert with ER oxidoreductin one to catalyze the formation of disulfide bonds. In this process, molecular oxygen is the ultimate electron acceptor, yielding one H_2_O_2_ molecule for every disulfide bond formed (Tu and Weissman, 2004). ER could be a better candidate for continuous H_2_O_2_ production (Rashdan and Pattillo, 2020) than POR. In this context, it is essential to note that ER is the compartment where ferrostatin-1 accumulates to combat ferroptosis (Gaschler et al., 2018).

#### 4.4.3 HMOX-1 inhibition in ferroptosis

The primary physiological role of HMOX-1 is heme clearance since the catalytic efficiency of heme exceeds that of free ferrous iron in generating ROS. Hence, depending on the context, HMOX-1 may play a dual role in ferroptosis. The number of reports on the benefits of HMOX-1 inhibition in various ferroptosis scenarios exceeds those on the protective effects of HMOX-1 expression. Even for intracerebral hemorrhage, HMOX-1 inhibition has demonstrated beneficial effects on outcome measures (Huang et al., 2024; Wang et al., 2024). HMOX-1 mediates inflammatory response in microglia of aged wild-type mice linked to iron deposits, oxidative stress, ferroptosis, and cognitive decline (Fernandez-Mendivil et al., 2021). HMOX-1 mediates ferroptosis in oxidative stress-mediated retinal pigment epithelium degeneration, and its inhibition results in substantial recovery of the retinal structure and visual function (Tang Z. et al., 2021). A protective effect of HMOX-1 inhibition is observed for erastin-induced ferroptosis in HT-1080 fibrosarcoma cells (Kwon et al., 2015)**.** It is important to mention that in all the above-cited papers, HMOX-1 activity has been inhibited with Zn-protoporphyrin (ZnPP), which targets not only HMOX-1 but also all heme sensor proteins. Among those, transcription factors Bach1 and Bach2 occupy the central place. They contain a heme sensor domain, which, upon heme or protoporphyrin binding, causes a conformational change in Bach1/2 to compromise its repression on antioxidant response elements (ARE) in DNA coding for proteins and enzymes of antioxidant defense. ZnPP is a known activator of the genetic antioxidant program that supports cell survival under oxidative stress conditions. Therefore, in the absence of specific inhibitors targeting HMOX-1 and Bach1/2 separately, the feasibility of employing a pharmacological strategy to investigate the role of HMOX-1 inhibition in protection against ferroptosis is impossible.

### 4.5 Ferrostatin as a potential therapeutic intervention

Ferrostatin’s ability to combat ferroptosis is primarily attributed to its radical neutralizing capacity to eliminate initial alkoxyl radicals formed in non-enzymatic ferrous ion-dependent lipid peroxidation reaction (Miotto et al., 2020). Although not yet approved for clinical use, ferrostatin and related compounds are under investigation for their potential to mitigate the progression of neurodegenerative diseases and other conditions associated with ferroptosis. (Skouta et al., 2014; Anthonymuthu et al., 2021). Ferrostatin may provide neuroprotection by preventing ferroptosis-induced neuronal death, potentially slowing down the advancement of the disease. In preclinical studies, ferrostatin and similar compounds have demonstrated efficacy in AD, PD (Wang Y. et al., 2023), and intracerebral hemorrhage models (Li et al., 2017). However, a derivative of ferrostatin (with an additional N-benzyl substitution at the ferrostatin-1 ortho-phenylene-diamine group) that protects against Friedreich’s ataxia (Cotticelli et al., 2019) and cerebral ischemia/reperfusion seemingly operates by activating Nrf2 signaling (Chen et al., 2023b). In the spinal cord injury model, ferrostatin-1 also works through the activation of Nrf2 (Zhou et al., 2023). In general, the ortho-phenylene-diamine scaffold in ferrostatin, when oxidized in the presence of metal traces, generates an oxidant (substituted 2,3-diamino phenazine) that could drive activation of Nrf2 by chemically modifying its inhibitor protein, Kelch-like ECH-associated protein 1 (Keap-1) (see section 4.6 below).

### 4.6 Nrf2 activation and Bach1 inhibition as a potential therapeutic approach

Protection against ferroptosis is strongly associated with the genes targeted by Nrf2, a key transcription factor that plays a pivotal role in maintaining cellular redox homeostasis. Nrf2 belongs to the CNC-bZIP family and orchestrates the expression of antioxidant and cytoprotective genes (Shakya et al., 2023). The Nrf2-Keap1 pathway is a strategic coordinator in the cellular defense against ferroptosis. This pathway effectively detects changes in cellular oxidative stress and triggers the expression of a wide range of Nrf2-regulated genes to counteract the initial stressor of ferroptosis. When exposed to oxidative or electrophilic stress, cysteine residues in Keap1 are modified, leading to its inactivation and subsequent Nrf2 accumulation. Nrf2 then translocate to the nucleus and binds to the antioxidant response element (ARE), promoting the expression of a gene battery that combats oxidative stress and cell death (Anandhan et al., 2020; Lane et al., 2021; Lane et al., 2023). Activation of Nrf2 enhances antioxidant defense mechanisms and modulates iron homeostasis directly. For instance, by increasing ferritin levels, a protein that stores iron, Nrf2 can sequester excess iron and mitigate its involvement in lipid peroxidation. This dual function of Nrf2, safeguarding against oxidative stress and iron-induced ferroptosis, underscores its potential as a therapeutic target (Dodson et al., 2019; Lane et al., 2023).

Conversely, Bach1, or BTB and CNC homology 1, a heme-responsive transcription factor, functions as a negative regulator of Nrf2 by competing for binding to the ARE. Bach1 and Nrf2 jointly regulate the availability of iron for specific cellular proteins. When Bach1 is highly active, or Nrf2 is less active, there is an excess of unstable iron, rendering cells more susceptible to ferroptosis (Nishizawa et al., 2020). This intricate balance between Nrf2 and Bach1 is crucial in cellular defenses against ferroptosis (Namgaladze et al., 2022; Nishizawa et al., 2023). Interventions, either through drugs or genetic approaches that inhibit Bach1’s function, can release the restraints on Nrf2, amplifying its protective responses. Combining Nrf2 activation with inhibition of Bach1 holds the potential for synergistic neuroprotection against ferroptosis.

**FIGURE 6:**
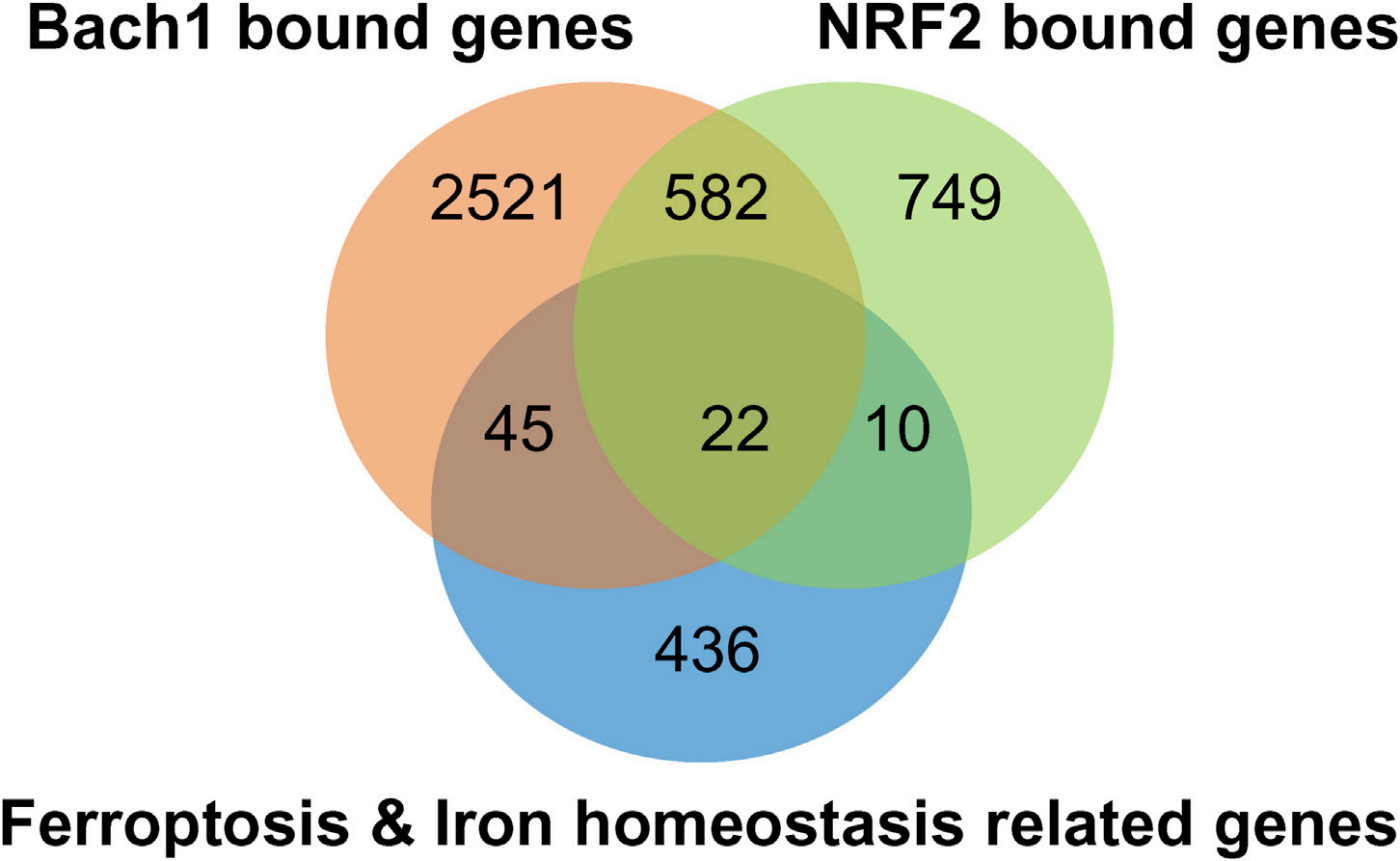
NRF2/BACH1 signaling axis modulates the expression of ferroptosis and iron homeostasis genes. Venn-diagram of ferroptosis and iron homeostasis genes shows them bound by NRF2 (light green) or Bach1 (orange) by ChIP-seq analysis. Ferroptosis- and iron homeostasis-related genes were extracted from MSigDB (Liberzon et al., 2011), overlaid with genes associated with Bach1 (Ahuja et al., 2021) or Nrf2 (Kobayashi et al., 2016) by ChIP-seq data.

**FIGURE 7:**
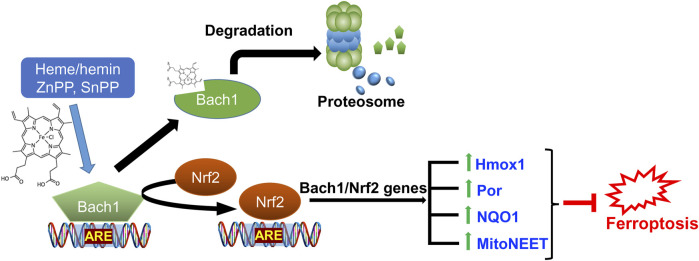
Role of Nrf2/Bach1 signaling axis in blocking ferroptosis. Bach1 works as a transcriptional repressor for ARE-site and competes with Nrf2. Upon heme or hemin, zinc protoporphyrin (ZnPP), and tin-protoporphyrin (SnPP) binding, Bach1 undergoes a conformational change, leading to its exit from the nucleus and subsequent degradation by the proteasome. The activation of Nrf2 results in the expression of hundreds of genes in antioxidant defense and iron regulation programs, including those directly implicated in counteracting ferroptosis (listed in Table 1).

Chromatin Immunoprecipitation (ChIP)-sequencing analysis of Bach1 and Nrf2 genes, which overlap with critical players in ferroptosis, reveals 22 common genes, as depicted in (Figure 6). These genes are involved in various pathways, as outlined in (Table 1). In addition to 22 common genes, 10 genes bound by Nrf2 (but not Bach1) are implicated in ferroptosis, along with 45 genes bound by Bach1 (but not Nrf2). In other words, Bach1 binds to more genes related to ferroptosis than Nrf2 does. Therefore, Bach1 is just as crucial for the ferroptosis pathway as Nrf2. Genes regulated by Nrf2 are vital molecular players in suppressing and preventing ferroptosis. At the same time, Bach1 interferes with Nrf2 binding to several gene targets (Figure 7). This interference extends to genes involved in glutathione synthesis enzymes (Gclm and Gclc), selenocysteine enzyme thioredoxin reductase 1 (Txnrd1), and proteins and enzymes related to iron homeostasis, such as heme oxygenase-1 (HMOX-1/POR) (Figure 7; Table 1). These genes are essential for combating lipid peroxidation and preventing ferroptosis (Lane et al., 2021; Yan et al., 2023). Mitochondrial aconitase (ACO2), the target for both Nrf2 and Bach1, serves as a redox sensor in the tricarboxylic acid cycle due to the high sensitivity of its active site ISC towards ROS. ACO2 is particularly susceptible to oxidation during aging and mitochondrial dysfunction. It is vital to replenish this enzyme, as it plays a pivotal role in maintaining the performance of the TCA cycle.

Another set of targets shared by Nrf2 and Bach1 is associated with lipid synthesis/storage and inflammatory reactions catalyzed by heme-containing enzymes. Neuroinflammation is a common feature of many neurodegenerative diseases, and activating Nrf2 has been shown to alleviate oxidative stress and neuroinflammation in mouse models of AD and PD. Therefore, combining Nrf2 activation with Bach1 inhibition represents a promising strategy to inhibit the inflammatory cascade and shield neurons from the detrimental effects of chronic inflammation.

The endosomal system is indispensable for normal brain development and function. Two notable endosomal targets relevant to insulin resistance and type 2 diabetes are the Zfyve28 and Exoc6 genes (as listed in Table 1). It is well-known that brain insulin resistance plays an essential role in AD development and progression (Sedzikowska and Szablewski, 2021). Regulation of Zfyve28 by Notch is well-documented (Yu et al., 2023), but its plausible link to Nrf2/Bach1 (as indicated in Table 1) is novel. Nrf2 and Bach1 share common targets in endo/exocytosis, neurogenesis, and protein degradation pathways (such as the TET1 enzyme, ubiquitin b, and c, as shown in Table 1). However, the interdependence of Nrf2/Bach1 effects on these targets in the context of neurodegenerative diseases needs to be further explored. Notably, Nrf2 and Bach1/2 transcription factors depend on each other and are associated with ferroptosis (as indicated in Table 1). There are no published studies regarding Bach1 and Bach2’s involvement in mediating ferroptosis in neurodegenerative diseases. Considering the data in Table 1, further research into the role of Bach1 and Bach2 in ferroptosis in neurodegenerative diseases of various etiology is warranted.

## 5 Conclusion and future perspectives

Various cellular metabolites, enzymes, and biological pathways work in tandem in the execution of ferroptosis. Although its role in cancer is now well-established, there is a growing body of evidence that ferroptosis plays a significant role in neurodegenerative diseases, but several unresolved questions remain. One of the challenges in investigating ferroptosis in cancer tissues and cell culture models is that the metabolic, genetic, and epigenetic changes may mask the accurate step-by-step mechanism of initiation and progression. On top of this, studying the process in cell cultures and tissues will always be overridden by the documented “wave” of ferroptosis propagation (Riegman et al., 2020), the spreading of a cell swelling effect through cell populations in a lipid peroxide (Nishizawa et al., 2021) and iron-dependent manner. In cell populations with a propagation wave, the inhibition studies on ferroptosis will always point towards a critical role of free iron. Ferroptosis models using cancer cell lines with common ferroptosis inducers may be too harsh and deceiving to elucidate sequential mechanisms working in neurodegeneration. In this respect, studying the onset of ferroptosis in cellular models of the neuronal origin of familial cases may give a better picture of the precise association between the underlying disease mechanisms leading up to ferroptosis. Even though we are still too far from understanding the details of ferroptotic pathways in neurodegenerative diseases, based on existing data, we are confident that the Bach1/Nrf2 signaling axis is one of the central targets for most forms of neurodegeneration. Such belief stems from the critical role played by Nrf2 targets in protection against ferroptosis and the need to relieve Bach1 repression to achieve the full therapeutic benefits of Nrf2 activation in neurodegenerative diseases (Ahuja et al., 2022; Hushpulian et al., 2024). Moreover, inhibiting Bach1 may uncover yet unknown additional benefits besides Nrf2 activation since Bach1 has significantly higher intersections with ferroptosis genes than Nrf2 (Figures 6, 7; Table 1). Targeting Nrf2 activation is an established and promising avenue for preventing/delaying the onset of neurodegeneration. Most of the existing treatments for neurodegenerative diseases aim to manage symptoms due to a lack of effective disease-modifying therapies. Given the significant contribution of ferroptosis in the pathophysiological processes driving neurodegenerative diseases, Bach1/Nrf2-based therapies may offer a disease-modifying approach by targeting the underlying disease mechanisms. The successful application of novel Bach1 inhibitors as therapeutic agents in preclinical animal models of PD further supports this viewpoint (Ahuja et al., 2021). Future development and optimization of this class of small molecule therapeutics will likely yield more effective and hopefully disease-modifying therapies targeting ferroptosis for neurodegenerative diseases.

## Figures and Tables

**Table:** 

**αS**	alpha-synuclein
**4-HNE**	4-hydroxynonenal
**ACO2**	Aconitase 2
**ACSL4**	Acyl-CoA Synthetase Long-Chain Family Member 4
**AD**	Alzheimer’s disease
**AIFM2**	Apoptosis-inducing factor mitochondrial 2
**ALS**	Amyotrophic lateral sclerosis
**AMPK**	AMP-activated protein kinase
**APOE**	Apolipoprotein E
**APP**	β-Amyloid Precursor Protein
**ARE**	Antioxidants response element
**Aβ**	Amyloid-beta
**Bach1**	Broad complex-tramtrack-bric-a-brac (BTB) and cap ‘n’ collar (CNC) homolog 1
**BBB**	Blood Brain Barrier
**BECN1**	Beclin 1
**BH4**	Tetrahydrobiopterin
**CBS**	Cystathionine β-synthase
**CDIS**	CDGSH iron-sulfur domain
**CoQ10**	Coenzyme Q10
**CSE**	Cystathione γ-lyase
**DHODH**	Dihydroorotate dehydrogenase
**DMT1**	Divalent Metal Transporter 1
**Drp1**	Dynamin-related protein 1
**ER**	Endoplasmic reticulum
**ERK**	Extracellular signal-regulated kinase
**FSP**	Ferroptosis suppressor protein 1
**FTH**	Ferritin Heavy Chain
**FTL**	Ferritin Light Chain
**gGCS**	gamma-glutamyl-cysteine synthetase
**GCH1**	Guanosine triphosphate cyclohydrolase I
**GPX4**	Glutathione Peroxidase 4
**GSEA**	Gene set enrichment analysis
**GSH**	Glutathione
**GSH/GSSG**	Reduced glutathione/oxidized glutathione ratio
**G6PD**	Glucose-6-phosphate dehydrogenase
**HD**	Huntington’s disease
**HIF1**	Hypoxia-inducible factor 1
**HMOX-1**	Heme Oxygenase-1
**IMM**	Inner Mitochondrial Membrane
**IRE**	Iron Response Element
**IRP**	Iron Regulatory Proteins
**ISC**	iron-sulfur cluster
**JAK-STAT**	Janus kinase signal transducer and activator of transcription
**Keap1**	Kelch-like-ECH-associated protein 1
**LIP**	Labile Iron Pool
**LOOH**	Lipid hydroperoxides
**LOX**	Lipoxygenases
**LPCAT3**	Lysophosphatidylcholine Acyltransferase 3
**mNT**	mitoNEET
**MAPK**	Mitogen-activated protein kinase
**MDA**	Malondialdehyde
**NADPH**	Nicotinamide adenine dinucleotide phosphate
**NCOA4**	Nuclear Receptor Coactivator 4
**NOS**	Nitric oxide synthases
**NQO1**	NAD(P)H quinone oxidoreductase 1
**Nrf2**	Nuclear factor erythroid 2-related factor 2
**OMM**	Outer Mitochondrial Membrane
**PAK1**	p21-activated kinase 1
**PCBP1**	Poly (rC)-binding protein
**PD**	Parkinson’s disease
**PE**	Phosphatidylethanolamine
**PEBP1**	Phosphatidylethanolamine-Binding Protein 1
**PHDs**	Prolyl Hydroxylases
**PI3K**	Phosphatidylinositol 3-kinase
**PINK1**	PTEN-induced kinase 1
**POR**	Cytochrome P450 oxidoreductase
**PUFA**	Polyunsaturated fatty acids
**RAC1**	Ras-related C3 botulinum toxin substrate 1
**RCD**	Regulated cell death
**ROS**	Reactive Oxygen Species
**RSL3**	RAS-selective lethal
**SN**	Substantia Nigra
**SNpc**	Substantia Nigra pars compacta
**STEAP3**	Six-transmembrane epithelial antigen of prostate 3
**TF**	Transferrin
**TfR**	Transferrin receptor
**TXNRD1**	Thioredoxin reductase 1
**UTR**	untranslated region
**VDAC**	Voltage-dependent anion channel
**VHL**	Von Hippel Lindau
**ZnPPP**	Zn-protoporphyrin

## Supplementary material

SUPPLEMENTARY FIGURE S1 Model structure of VDAC2 and its complexes with mNT. **(A)** The model structure of the VDAC2 from Alphafold (https://alphafold.ebi.ac.uk/entry/P45880) as schematic solid ribbon. Cysteine residues are shown in yellow. **(B)** Possible orientation of mitoNEET monomer inside VDAC2. Both are shown as hydrophobic surfaces. **(C)** Possible orientation of mitoNEET dimer inside VDAC2. The mitoNEET dimer is shown as a hydrophobic surface, and VDAC2 is a schematic solid ribbon. Docking is prepared using the open-access GRAMM docking server. Structures with PDB IDs 4BUM and 3EW0 were used for VDAC2 and mitoNEET, respectively.
