# Supplementary figures and images for: A critical appraisal of ferroptosis in Alzheimer’s and Parkinson’s disease: new insights into emerging mechanisms and therapeutic targets
Source: Front Pharmacol. 2024 Jul 8;15:1390798. doi: 10.3389/fphar.2024.1390798 (PMC11260649; doi:10.3389/fphar.2024.1390798)

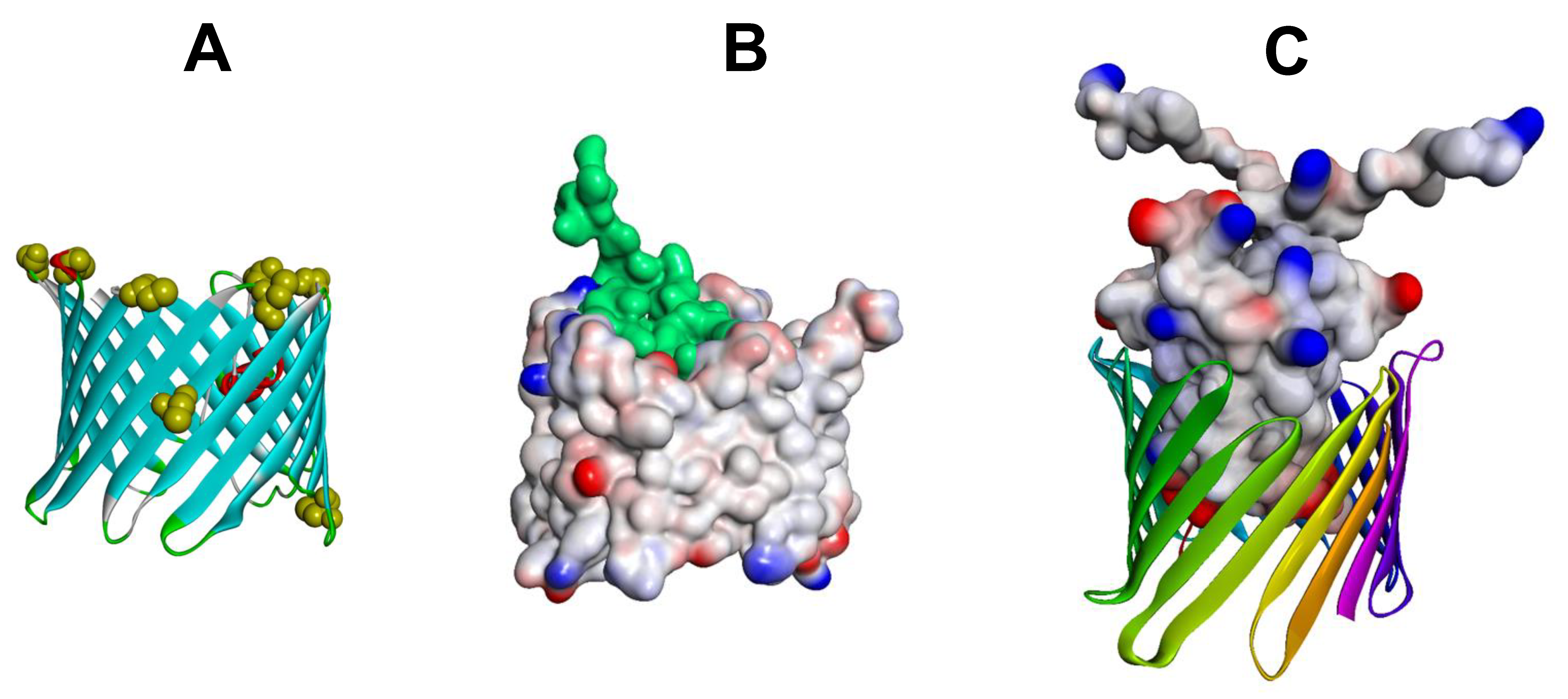

Supplement: Supplementary file 1 [file Image1.TIF]
